# Oxysterol levels and metabolism in the course of neuroinflammation: insights from in vitro and in vivo models

**DOI:** 10.1186/s12974-018-1114-8

**Published:** 2018-03-09

**Authors:** Valentin Mutemberezi, Baptiste Buisseret, Julien Masquelier, Owein Guillemot-Legris, Mireille Alhouayek, Giulio G. Muccioli

**Affiliations:** Bioanalysis and Pharmacology of Bioactive Lipids Research Group, Louvain Drug Research Institute (LDRI), Université catholique de Louvain (UCL), Av. E. Mounier, 72 (B1.72.01), 1200 Bruxelles, Belgium

**Keywords:** Hydroxycholesterol, Quantification, M1 polarization, M2 polarization, Cytochrome, Cytokines

## Abstract

**Background:**

Oxysterols are cholesterol derivatives that have been suggested to play a role in inflammatory diseases such as obesity, atherosclerosis, or neuroinflammatory diseases. However, the effect of neuroinflammation on oxysterol levels has only been partially studied so far.

**Methods:**

We used an HPLC-MS method to quantify over ten oxysterols both in in vitro and in vivo models of neuroinflammation. In the same models, we used RT-qPCR to analyze the expression of the enzymes responsible for oxysterol metabolism. Using the BV2 microglial cell line, we explored the effect of lipopolysaccharide (LPS)-induced (M1-type) and IL-4-induced (M2-type) cell activation on oxysterol levels. We also used LPS-activated co-cultures of mouse primary microglia and astrocytes. In vivo, we induced a neuroinflammation by administering LPS to mice. Finally, we used a mouse model of multiple sclerosis, namely the experimental autoimmune encephalomyelitis (EAE) model, that is characterized by demyelination and neuroinflammation.

**Results:**

In vitro, we found that LPS activation induces profound alterations in oxysterol levels. Interestingly, we could discriminate between control and LPS-activated cells based on the changes in oxysterol levels both in BV2 cells and in the primary co-culture of glial cells. In vivo, the changes in oxysterol levels were less marked than in vitro. However, we found in both models increased levels of the GPR183 agonist 7α,25-dihydroxycholesterol. Furthermore, we studied in vitro the effect of 14 oxysterols on the mRNA expression of inflammatory markers in LPS-activated co-culture of microglia and astrocytes. We found that several oxysterols decreased the LPS-induced expression of pro-inflammatory markers.

**Conclusions:**

These data demonstrate that inflammation profoundly affects oxysterol levels and that oxysterols can modulate glial cell activation. This further supports the interest of a large screening of oxysterol levels when studying the interplay between neuroinflammation and bioactive lipids.

**Electronic supplementary material:**

The online version of this article (10.1186/s12974-018-1114-8) contains supplementary material, which is available to authorized users.

## Background

Inflammation as a primary or secondary component in neuropathology is proving to be an intrinsic part of the pathophysiology of neurodegenerative diseases. This inflammatory component has been suggested to be the common link between many of these diseases such as Alzheimer’s disease, Parkinson’s disease, and multiple sclerosis [1–3]. Different hypotheses have been explored in order to better understand the exact role of inflammation in the pathophysiology of neurodegenerative diseases and extend the number of potential therapeutic targets [2, 4, 5]. One of the promising axes in this field is the implication of bioactive lipids.

Oxysterols, which are oxidized derivatives of cholesterol, are one family of bioactive lipids which have been suggested to play a role in inflammation and neuroinflammation [6–10]. Indeed, some authors suggested that the levels, or the ratio, of 24(*S*)-hydroxycholesterol (24(*S*)-OHC), the most abundant oxysterol in the brain, and 27-OHC in plasma and cerebrospinal fluid could be used as biomarkers to monitor evolution or treatment efficacy in neurodegenerative and neuroinflammatory diseases [11–14]. In terms of effects, 27-OHC and 7α,25-di-OHC have been described as pro-inflammatory, while other oxysterols, such as 22(*R*)-OHC, showed anti-inflammatory effects [6, 8]. However, determining the pathways responsible for the observed effects remains a challenge as an increasing number of molecular targets mediating the effects of oxysterols have been described [15]. Probably, the most well known are the nuclear receptors, liver X receptors (LXR), and retinoic acid-related orphan receptors (ROR) [16–18]. Other targets have been also identified such as the G protein-coupled receptor GPR183 (also known as EBI2) and CXCR2 [19–21].

Surprisingly, most of the studies with oxysterols focus on a single oxysterol and do not really take into account the existence of a multitude of species in the oxysterol family or the discovery of new targets [22, 23]. Hence, there is a need for further studies in order to better understand the effects and roles of oxysterols in neuroinflammation.

In this study, we analyzed the variations of endogenous levels of numerous oxysterols in neuroinflammatory settings, both in vitro and in vivo. In in vitro settings, we used the BV2 microglial-like cell line as well as primary co-cultures of microglia and astrocytes. In vivo, we analyzed oxysterol levels in an acute model of lipopolysaccharide (LPS)-induced inflammation in mice and in a murine model of multiple sclerosis, the experimental autoimmune encephalomyelitis (EAE) model. We also studied the effects of 14 oxysterols on LPS-induced activation of primary glial cells in vitro.

## Methods

### Oxysterol standards and reagents

4β-hydroxycholesterol (4β-OHC), 5α,6α-epoxycholesterol (5α,6α-epoxychol), 5α,6β-dihydroxycholesterol (5α,6β-di-OHC), 5β,6β-epoxycholesterol (5β,6β-epoxychol), 7α-hydroxycholesterol (7α-OHC), 7β–hydroxycholesterol (7β-OHC), 7α-hydroxycholestenone (7α-OHCnone), 7-ketocholesterol (7-ketochol), 22(*R*)-hydroxycholesterol (22(*R*)-OHC), 24(*S*)-hydroxycholesterol (24(*S*)-OHC), 25-hydroxycholesterol (25-OHC), 7α,25-dihydroxycholesterol (7α,25-di-OHC), 27-hydroxycholesterol (27-OHC), 7α,27-dihydroxycholesterol (7α,27-di-OHC), 24(*S*),25-epoxycholesterol (24(*S*),25-epoxychol), [25,26,26,26,27,27,27-^2^H_7_]4β-hydroxycholesterol (d_7_-4β-OHC), and [25,26,26,26,27,27,27-^2^H_7_]24(*R/S*)-hydroxycholesterol (d_7_-24-OHC) were purchased from Avanti polar lipids (USA). HPLC-MS grade dichloromethane, isopropanol, hexane, and methanol were purchased from VWR Belgium. Butylated hydroxytoluene (BHT) and ethylenediaminetetraacetic acid (EDTA) were purchased from Sigma Aldrich. Lipopolysaccharides (LPS; E.coli O55:B5) and mouse recombinant IL-4 were purchased from Sigma Aldrich as well.

### BV2 cell line culture

BV2 microglial-like cells (a generous gift from E. Hermans, IONS, UCL, Belgium) were grown in high-glucose DMEM medium with 10% FBS and antibiotics (100 U/mL penicillin and 100 μg/mL streptomycin) and were cultured under standard conditions (37 °C in a humidified 5% CO_2_ incubator). The cells were seeded overnight into 24-well plates (2.5 × 10^5^ cells per well) for RNA extraction and qPCR assay, and into 100 mm dishes (10^7^ cells per dish) for oxysterol quantification. The experiments were performed the next morning. For experiments investigating the effect of LPS-induced activation of BV2 cells (M1 activation) on oxysterol levels, medium was changed and fresh culture medium with 1% FBS and containing 100 ng/mL of LPS was added for 4, 8, 16, and 24 h [24]. For experiments investigating the effect of IL-4-induced activation of BV2 cells (M2 activation) on oxysterol levels, the medium was changed and fresh culture medium containing 10 U/mL of IL-4 was added for 12 and 24 h [25, 26]. For all experiments, a control condition was performed, where cells were seeded concomitantly but were only incubated with vehicle in the absence of LPS or IL-4 for the determined time depending on the experiments.

### Animal experiments

All studies involving animals are in compliance with the European Directive 2010/63/EU, transformed into the Belgian Law of May 29, 2013, regarding the protection of laboratory animals (agreement number LA1230635) and were approved by the Université catholique de Louvain animal committee (2014/UCL/MD/001). Mice were housed in a controlled environment with a 12-h light-dark cycle and access to food and water ad libitum.

#### Primary co-culture of microglia and astrocytes

Mice pups (post-natal day 2–3) were euthanized, the brain recovered, and their cerebral cortices dissected. Following mechanical dissociation of tissues by several sequences of pipetting and sedimenting, then centrifugation, the cells were resuspended in DMEM-F12 media (containing 10% FBS, 100 U/mL of penicillin, and 100 μg/mL of streptomycin) and seeded in poly-lysine pre-coated flasks (2 cortices/flask). The cells were incubated for 2 weeks with two media changes at days 5 and 10. After 14 days in culture, the cells were seeded overnight in poly-lysine pre-coated 24-well plates for RNA extraction and qPCR assay, and into 100 mm dishes for oxysterol quantification. The experiments were performed the next morning [27].

For experiments investigating the effect of LPS-induced activation of this co-culture of primary glial cells on oxysterol levels, the medium was changed and fresh culture medium containing 100 ng/mL of LPS was added for 8 and 24 h.

For experiments investigating the effect of oxysterols on LPS-induced activation of the primary glial co-culture, cells were incubated with fresh culture medium containing 10 μM of the oxysterols of interest, and 100 ng/mL of LPS was added 1 h later, for 8 h. Of note, 10 μM is a classically used concentration for bioactive lipids testing in vitro, including for oxysterols [28–30].

For all experiments, a control condition was made alongside, where cells were seeded and incubated with vehicle only (DMSO, 0.2%), in the absence of LPS, for the determined time depending on the experiments.

#### LPS-induced inflammation

Six- to eight-week-old male C57BL/6 mice (Charles River Laboratories) were housed under standard conditions and supplied with drinking water and food ad libitum. LPS (300 μg/kg, i.p) was administered to mice, in a saline containing 0.1% Tween 80 vehicle [31]. After either 4 or 8 h, mice (7 per group) were deeply anesthetized before sacrifice. The tissues were rapidly recovered and snap frozen into liquid nitrogen before storing them at − 80 °C until oxysterol quantification was performed.

#### Experimental autoimmune encephalomyelitis

Experimental autoimmune encephalomyelitis (EAE) was induced in C57Bl/6 female mice (8 weeks of age) as the prevalence of the disease is higher in women and the susceptibility of female mice to EAE is higher [32, 33]. Mice were immunized with a subcutaneous injection in the flanks with 200 μg of rMOG35-55 in complete Freund’s adjuvant (CFA) containing 8 mg/mL of Mycobacterium tuberculosis H37 on day 0. Control mice received CFA without rMOG35-55. Mice received 250 ng of pertussis toxin (PTX) i.p. on days 0 and day 2. Weight and clinical score were recorded daily using the following scale for the clinical score: 0 = no clinical signs, normal mouse; 1 = flaccid tail; 2 = flaccid tail and hind limb weakness; 3 = partial hind limb paralysis; 4 = complete hind limb paralysis; 5 = moribund state or death. Mice were sacrificed when the clinical score was either 0.5–1 or 3–4.

### Oxysterol quantification

Oxysterols were analyzed by using a validated HPLC-MS method [34]. Briefly, after the incubation period, the media were removed and 2 mL of fresh PBS (phosphate-buffered saline) added to the dishes. Then, the cells were scraped and transferred into glass vials containing dichloromethane-methanol and internal standards (d_7_-4β-OHC and d_7_-24-OHC) for extraction. BHT (10 μg/vial) and EDTA (20 ng/vial) were added to the mixture to avoid artifactual generation of oxysterols due to cholesterol oxidation during the procedure. The final proportion of solvent in the extraction mixture was 8:4:2 (v/v/v) dichloromethane-methanol-PBS. For the tissues (the liver, brain, and spinal cord), the frozen sections were directly homogenized in a glass vial containing dichloromethane. Then, internal standards, BHT, and EDTA were added and extraction mixture completed by methanol and bi-distilled water in order to obtain the same proportions as above. The vials were then shaken vigorously and sonicated for 10 min at 4 °C. After centrifugation, the organic phase was recovered and evaporated under a nitrogen stream. Oxysterols were pre-purified by solid-phase extraction (SPE) using silica as stationary phase. Hexane-isopropanol was used to remove cholesterol from the samples, after which, oxysterols were eluted by increasing the proportion of isopropanol. The oxysterol fraction was analyzed by HPLC-MS using a LTQ-Orbitrap mass spectrometer (ThermoFisher Scientific) coupled to an Accela HPLC system (ThermoFisher Scientific). Analyte separation was achieved using a C-18 Supelguard pre-column and a kinetex LC-18 column (5 μm, 4.6 × 150 mm) (Phenomenex). Mobile phases A and B were composed of MeOH-H_2_O-acetic acid 75:24.9:0.1 (*v*/*v*/*v*) and MeOH-acetic acid 99.9:0.1 (*v*/*v*), respectively. The gradient (0.4 mL/min) was designed as follows: transition from 100% A to 100% B linearly over 15 min, followed by 10 min at 100% B and subsequent re-equilibration at 100% A. We performed mass spectrometry analysis in the positive mode with an APCI ionization source. For data acquisition and processing, the Xcalibur® software (ThermoFisher Scientific) was used. Oxysterols were quantified using internal standards, d_7_-24-OHC for those oxidized on the lateral side chain and d_7_-4β-OHC for those which are oxidized on the sterol backbone. Calibration curves were prepared using the same conditions, including the liquid extraction and the SPE [34].

### Real-time qPCR

At the end of the incubation period, media was removed and TriPure reagent was added to each well of the tissue culture plates. The plates were then stored at − 80 °C for later assessment. Total RNA was extracted using the TriPure reagent according to the manufacturer’s instructions. cDNA was synthesized using a reverse transcription kit (Promega corporation) from 1 μg of total RNA. qPCR was performed using a STEPone PLUS instrument and software (Applied Biosystems) as previously described [35]. Products were analyzed by performing a melting curve at the end of the PCR reaction. Data are analyzed with the ΔΔCt method using the 60S ribosomal protein L19 (RPL19) as a reference gene. RPL19 mRNA expression was not affected by any of the treatments. Primer sequences used are given in Table 1.

**Table 1:** Sequence of the primers used in this study

Gene	Product	Forward primer (5′ to 3′)	Reverse primer (5′ to 3′)
Arg1	Arginase 1	GGTTCTGGGAGGCCTATCTT	TGAAAGGAGCCCTGTCTTGT
Ccl3	MIP-1α	AGATTCCACGCCAATTCATC	CTCAAGCCCCTGCTCTACAC
Ch25h	Cholesterol-25-hydroxylase	CTGACCTTCTTCGACGTGCT	GGGAAGTCATAGCCCGAGTG
Chil3	YM-1	GCTTTTGAGGAAGAATCTGTGGA	AAGAGACTGAGACAGTTCAGGGAT
Cyp27a1	CYP27A1	GGCTACCTGCACTTCCT	CTGGATCTCTGGGCTCTTTG
Cyp7b1	CYP7B1	TAGGCATGACGATCCTGAAA	TCTCTGGTGAAGTGGACTGAAA
Ebp (D8D7I)	Cholesterol epoxide hydrolase (ChEH)	AGGGCTGGTTCTCTCTCTAC	CGACGAAGCTGTCACTAAGG
Hsd3b7	HSD3B7	CAGTCCACAGCCATCCCTACC	CGTGGGTCGAAGGGCACA
Hsd11b1	11β-HSD1	CCTTGGCTGGGAAAATGACCC	AGGACACAGAGAGTGATGGACA
Il1b	IL-1β	TCGCTCAGGGTCACAAGAAA	CATCAGAGGCAAGGAGGAAAAC
Il6	IL-6	ACAAGTCGGAGGCTTAATTACACAT	TTGCCATTGCACAACTCTTTTC
Mrc1	CD206	CCGTCTGTGCATTTCCATTC	TGCCAGTCAGTGGATCTTTGT
Ptgs2	COX-2	TGACCCCCAAGGCTCAAATAT	TGAACCCAGGTCCTCGCTTA
Rpl19	RPL19	GAAGGTCAAAGGGAATGTGTTCA	CCTTGTCTGCCTTCAGCTTGT
Tnf	TNF-α	AGCCCCCAGTCTGTATCCTT	GGTCACTGTCCCAGCATCTT

### Results expression and statistics software

Experiments were performed at least three times in triplicates for qPCR and quadruplicates for oxysterol quantification. The results are expressed as mean ± SEM. The data analysis was performed using GraphPad® prism 6. The data were analyzed with a One Way ANOVA followed by the Bonferroni multiple comparisons tests. When the data were not normally distributed, a Kruskall Wallis analysis was performed followed by the Dunn’s post-test. The partial least-squares discriminant analysis (PLS-DA) was performed using Multibase® 2015 (Numerical dynamics) and JMP Pro 12 at each time point of the study to discriminate the LPS-activated cells versus the control cells. The quality of the model was evaluated by the value of *R*^2^ and *Q*^2^. The former measures the goodness of fit while the latter measures the predictive ability of the model [36]. Finally, a trend was suggested when *p* value < 0.1.

## Results and discussion

Oxysterols have been suggested to play a role in neuroinflammation [6–8] and dysfunctions in cholesterol metabolism have been associated with neurodegenerative and neuroinflammatory diseases [37, 38]. However, few studies analyzed the effect of glial cell activation on oxysterol levels. Microglial cells are the early responders to inflammatory stimuli [25], therefore, we started this work by exploring the variation of oxysterol levels induced by LPS activation in the BV2 microglial cell line.

### Kinetic of oxysterol levels in LPS-activated BV2 cells

We activated BV2 cells, one of the most used microglial cell lines [24, 39, 40], with LPS (100 ng/mL), and we followed the variation of oxysterol levels at four different time points (4, 8, 16, or 24 h) [24, 31]. To assess BV2 cell activation, we measured the mRNA expression of several cytokines –IL-1β, IL-6, and TNF-α– classically associated with M1 polarization of macrophages. As expected, we found a time-dependent increase at the different time points of these pro-inflammatory markers in LPS-activated cells compared to control cells (Additional file 1: Figure S1A).

**Fig. 1:**
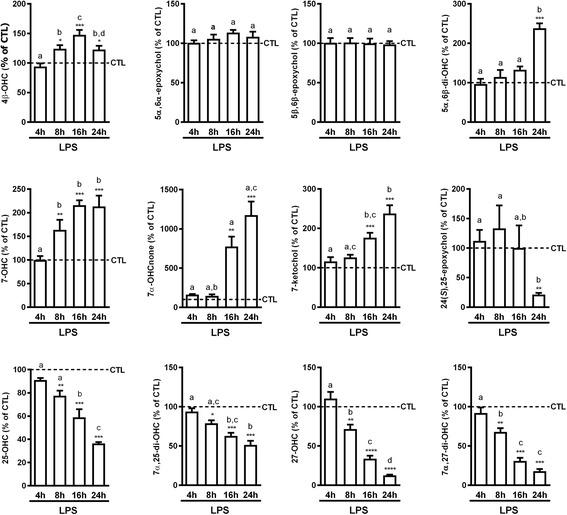
Kinetic of oxysterol variations in LPS-activated BV2 cells in comparison to control cells. 10^7^ cells were incubated in media with 1% FBS and containing LPS (100 ng/mL) or vehicle (CTL). Oxysterol levels were analyzed at four different time points: 4, 8, 16, and 24 h. The data are expressed as the mean ± SEM in percentage of their respective controls (CTL). ****p < 0.0001; ***p < 0.001; **p < 0.01; and *p < 0.05 for LPS-activated cells versus their respective controls. The different letters (a, b, c, d) indicate differences between the time points

We analyzed the oxysterols present in these cells and the effect of LPS-induced activation on their levels using a recently validated HPLC-MS method [34]. We found that 27-OHC is the most abundant oxysterol in this cell line (15 pmol/million cells), while the other oxysterols are present at lower levels (< 5 pmol/million cells) (Additional file 1: Figure S1B). For all the oxysterols analyzed, we found that 4 h of incubation with LPS did not induce apparent changes in oxysterol levels (Fig. 1). However, with increasing incubation periods, we could separate the oxysterols into three groups: (i) the two epoxides, 5α,6α-epoxychol and 5β,6β-epoxychol, were stable for the duration of the study; (ii) the levels of the other oxysterols oxidized on the sterol backbone were generally increased (e.g., 7-ketochol and 5α,6β-di-OHC), while (iii) the levels of the oxysterols oxidized on the side chain (e.g., 25-OHC and 27-OHC) were decreased in a time-dependent manner (Fig. 1). With the exception of 4β-OHC, which exhibited a peak at 16 h and a tendency to return to the control levels after 24 h, the variations in the levels of the other oxysterols were more marked at the later time points (i.e., after 24 h of LPS incubation).

**Fig. 2:**
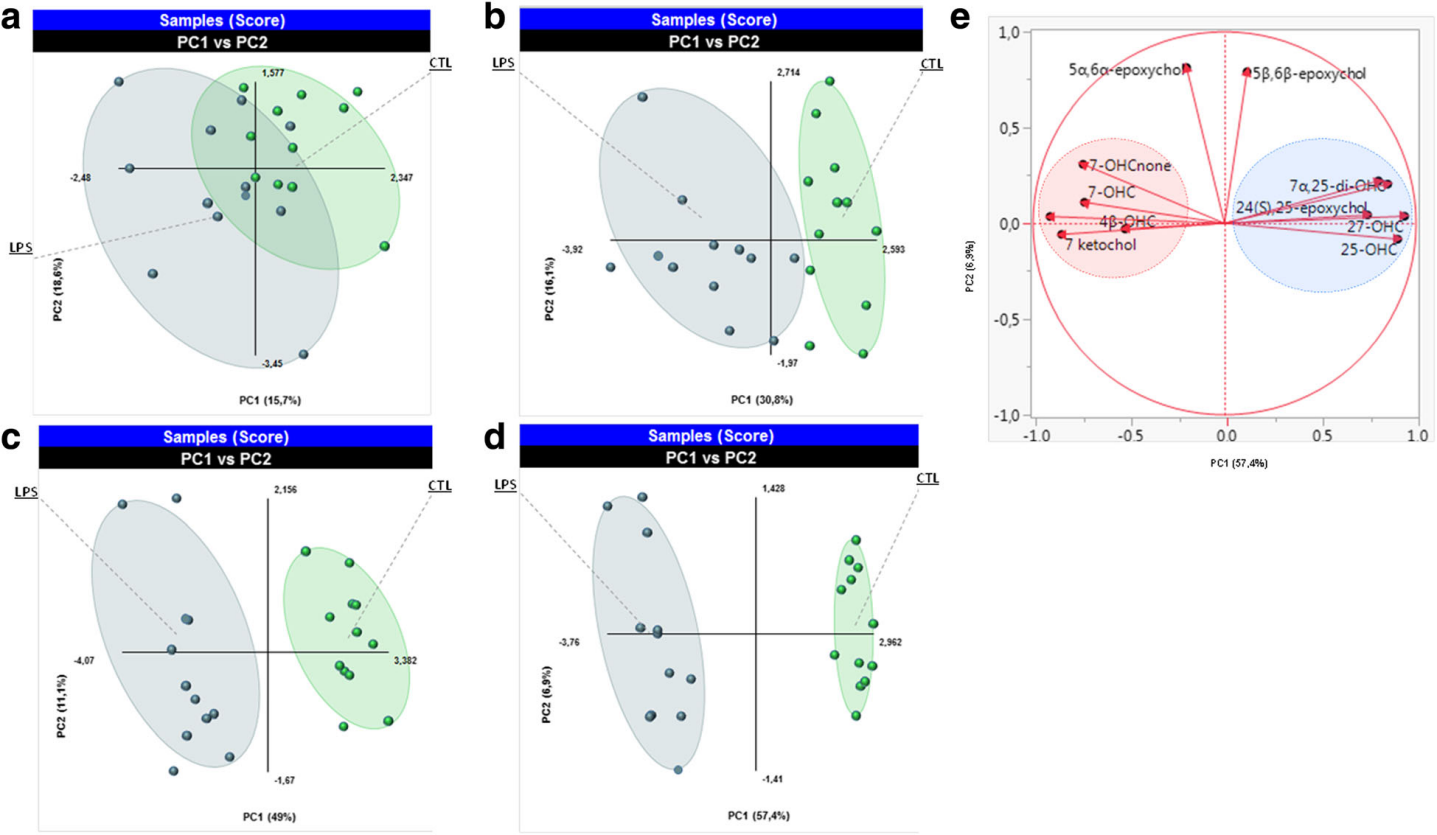
Multivariate analysis on all data in oxysterol levels variation in BV2 cells. The result of the partial least-squares discriminant analysis (PLS-DA). The score plot obtained at each time point of the kinetic **a** 4 h, **b** 8 h, **c** 16 h, and **d** 24 h are shown. Each dot on the graph represents a dish of cells. The quality of the model was assessed by the values of *R*^2^ and *Q*^2^. These values were 0.36 and 0.73, respectively, after 4 h; 0.76 and 0.63 after 8 h; 0.96 and 0.89 after 16 h; and 0.98 and 0.96 after 24 h. **e** The loading plot of different oxysterols at the 24 h time point

To determine whether these time-dependent changes in oxysterol levels induced by LPS would allow for distinguishing between control and LPS-treated cells, we performed a partial least-squares discriminant analysis (PLS-DA) with the data of each time point of the study (Fig. 2a–d). A significant separation between control and LPS-treated cells is apparent after 8 h of incubation, and this separation increases with longer incubation periods, allowing for discrimination between control and LPS-activated BV2 cells. The loading plot confirmed the observations mentioned above with, for instance, two main clusters at the 24-h time point, the first one contained oxysterols oxidized on the sterol backbone (4β-OHC, 5α,6β-di-OHC, 7-OHC, 7α-OHCnone, and 7-ketochol) which shared an increase of their levels in LPS-activated cells (Fig. 2e). The second cluster consists in oxysterols oxidized on the side chain or their metabolites (25-OHC, 7α,25-di-OHC, 27-OHC, and 7α,27-di-OHC) which are all decreased in LPS-activated cells in comparison to the control cells. Of note, while 24(*S*)-OHC is reported as the main OHC in the central nervous system, we did not detect it in the BV2 cells.

### Effect of LPS activation of BV2 cells on the mRNA expression of oxysterol-metabolizing enzymes

**Fig. 3:**
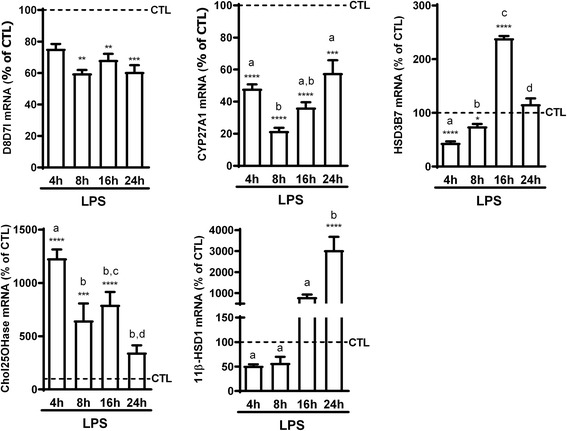
Kinetic of mRNA expression of oxysterol-metabolizing enzymes in LPS-activated BV2 cells compared to control cells. 2.5 × 10^5^ cells were incubated with LPS (100 ng/mL) or vehicle (CTL) for the indicated time points. mRNA was extracted and RT-qPCR performed for CYP27A1, cholesterol 25 hydroxylase (Chol25OHase), HSD3B7, 11β-HSD1, and the catalytic subunit of cholesterol epoxyde hydrolase (D8D7l). The data are expressed as the mean ± SEM in percentage of their respective CTL. ****p < 0.0001; ***p < 0.001; **p < 0.01; and *p < 0.05 for LPS-activated cells versus their respective controls. The different letters (a, b, c, d) indicate differences between the time points

To try and explain the variations we observed following activation of BV2 cells, we looked into the expression of the enzymes potentially involved in the metabolism of these oxysterols (Fig. 3).

Some oxysterols are produced via cholesterol oxidation induced by reactive oxygen species (ROS) [41], while others are produced following enzymatic reactions [15, 42, 43]. Some, such as 7-OHC (pool of 7α- and 7β-OHC) and 7-ketochol, are formed through both chemical oxidations by ROS and enzymatic processes [42]. LPS activation of macrophage-like cells generally leads to the production of ROS [44–46], which could explain the increase in the levels of these oxysterols. However, the impact of LPS-induced activation of the BV2 cells on mRNA expression of the oxysterol-metabolizing enzymes could also explain some of the variations we observed. Indeed, the expression of CYP27A1, which transforms 7-OHC and 7-ketochol was decreased upon LPS-induced activation (Fig. 3), in line with the increased levels of these oxysterols (Fig. 1). Furthermore, CYP27A1 is also responsible for the biosynthesis of 27-OHC [47]; therefore, the decrease in its mRNA expression is also in line with the decreased levels of 27-OHC and 7α,27-di-OHC (Fig. 1). Moreover, expression of HSD3B7 is increased after 16 h of LPS activation (Fig. 3) similarly to the levels of its product 7α-OHCnone (Fig. 1). However, mRNA expression of cholesterol-25-hydroxylase (Chol25OHase), the enzyme responsible for 25-OHC formation [48], and subsequently 7α,25-di-OHC production, was increased (Fig. 3), while the levels of these two oxysterols were decreased by LPS-induced activation of the cells. The increase in HSD3B7 which metabolizes 7α,25-di-OHC could be implicated in its decreased levels. Further studies will have to address if the decreased levels of 25-OHC and 7α,25-di-OHC are due to their metabolism into sulfated derivatives and cholestanoic acids, respectively.

On the other hand, the levels of two ROS-dependent oxysterols, 5α,6α-epoxychol and 5β,6β-epoxychol, are not increased. However, the levels of 5α,6β-di-OHC, of which the two epoxides are precursors, are increased. More intriguingly, this increase is in discordance with the decreased mRNA expression of the D8D7I (Fig. 3), the catalytic subunit of the cholesterol epoxide hydrolase, the enzyme responsible for the hydrolysis of the epoxycholesterols [49]. In line with the absence of 24(*S*)-OHC in these cells, we could not detect mRNA for CYP46A1, responsible for the synthesis of 24(*S*)-OHC.

Therefore, while mRNA expression of some of these enzymes (such as CYP27A1 and HSD3B7) could explain the variations in oxysterol levels we observed, some discrepancies remain probably due to post-transcriptional regulation of these enzymes and to the complex metabolic pathway of oxysterols and their derivatives.

### Variation of oxysterol levels in IL-4-activated BV2 cell line

Microglia present a plasticity in their polarization which leads to the coexistence of both M1 and M2 polarized cells in the response to inflammatory stimuli [50–52].

**Fig. 4:**
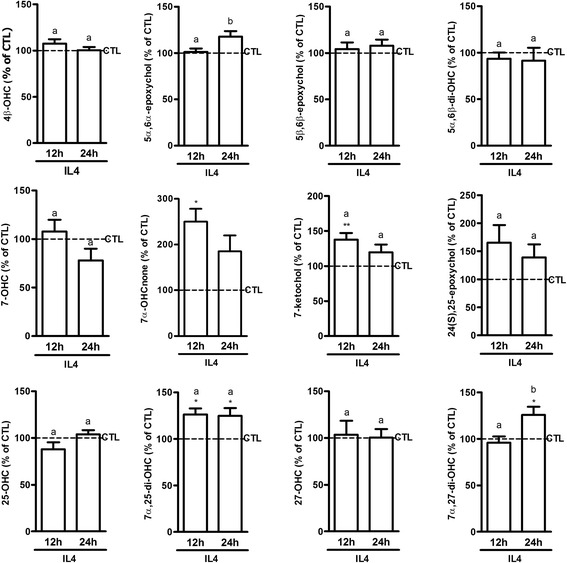
Oxysterol levels in IL-4-activated BV2 cells in comparison to control cells. 10^7^ cells were incubated in media with 1% FBS and containing 10 U/mL of interleukin 4 (IL-4) or vehicle (CTL). Oxysterol levels were analyzed at two time points: 12 and 24 h. The data are expressed as the mean ± SEM in percentage of their respective controls (CTL). **p < 0.01 and *p < 0.05 for LPS-activated cells versus their respective controls. The different letters (a, b) indicate differences between the time points

Similarly to what we did for the M1 polarization, we investigated the effect of a M2 polarization on oxysterol levels. Thus, we incubated BV2 cells with IL-4 (10 IU/ml) for 12 or 24 h, as typically reported, in order to induce a M2-type polarization [25, 26]. To assess the M2 phenotype, we measured the mRNA expression of arginase 1 and CD206, two common markers of M2 polarization, and found them increased after both 12 and 24 h of incubation with IL-4 (Additional file 1: Figure S1C). Contrasting with what we observed following LPS incubation, we observed only few variations in oxysterol levels after either 12 or 24 h of IL-4 incubation (Fig. 4). The only statistically significant variations were the increased levels of 7-OHCnone and 7-ketochol at 12 h, 7α,27-di-OHC after 24 h and finally 7α,25-di-OHC which was increased after both 12 and 24 h of incubation (Fig. 4). Moreover, there were little similarities when comparing these data to the data obtained in LPS-activated cells showing that M1 and M2 activation results in different changes in oxysterol levels.

### Variations of oxysterol levels in LPS-activated co-culture of primary microglia and astrocytes

One potential caveat of this approach is that BV2 cells, while widely used as a microglial-like cell line, might not model all the characteristics of in vivo microglial biology. [53] However, they show many similarities to primary microglia in their response to LPS [24] and offer the advantage of being stable in culture and excluding the need for animal use in early proof of concept studies. Moreover, there is interplay between different cell types in vivo, such as astrocytes, neurons, and microglia, as well as inputs from the periphery to the brain; therefore, even isolation of microglial populations would come with its inherent caveats. With this in mind, we sought to analyze the variations in oxysterol levels in a co-culture of mouse primary glial cells (containing microglia and astrocytes). This model, although lacking neuronal input, can be considered as more closely related to the in vivo situation where microglia interact with other glial cells in response to the inflammatory stimuli [54, 55]. We analyzed oxysterol levels in these glial cells after both 8 and 24 h of LPS incubation in order to be able to compare the results with those obtained on LPS-activated BV2 cells. Of note, we verified the activation state of glial cells upon LPS incubation by analyzing mRNA expression of cytokines (IL-1β, IL-6, and TNF-α) (Additional file 1: Figure S2A). When looking at the oxysterol levels in control cells, we found 7-ketochol and 7-OHC to be the most abundant oxysterols, whereas 27-OHC, which was the most abundant oxysterol in BV2 cells, is present at very low levels in these primary glial cells (Additional file 1: Figure S2B).

**Fig. 5:**
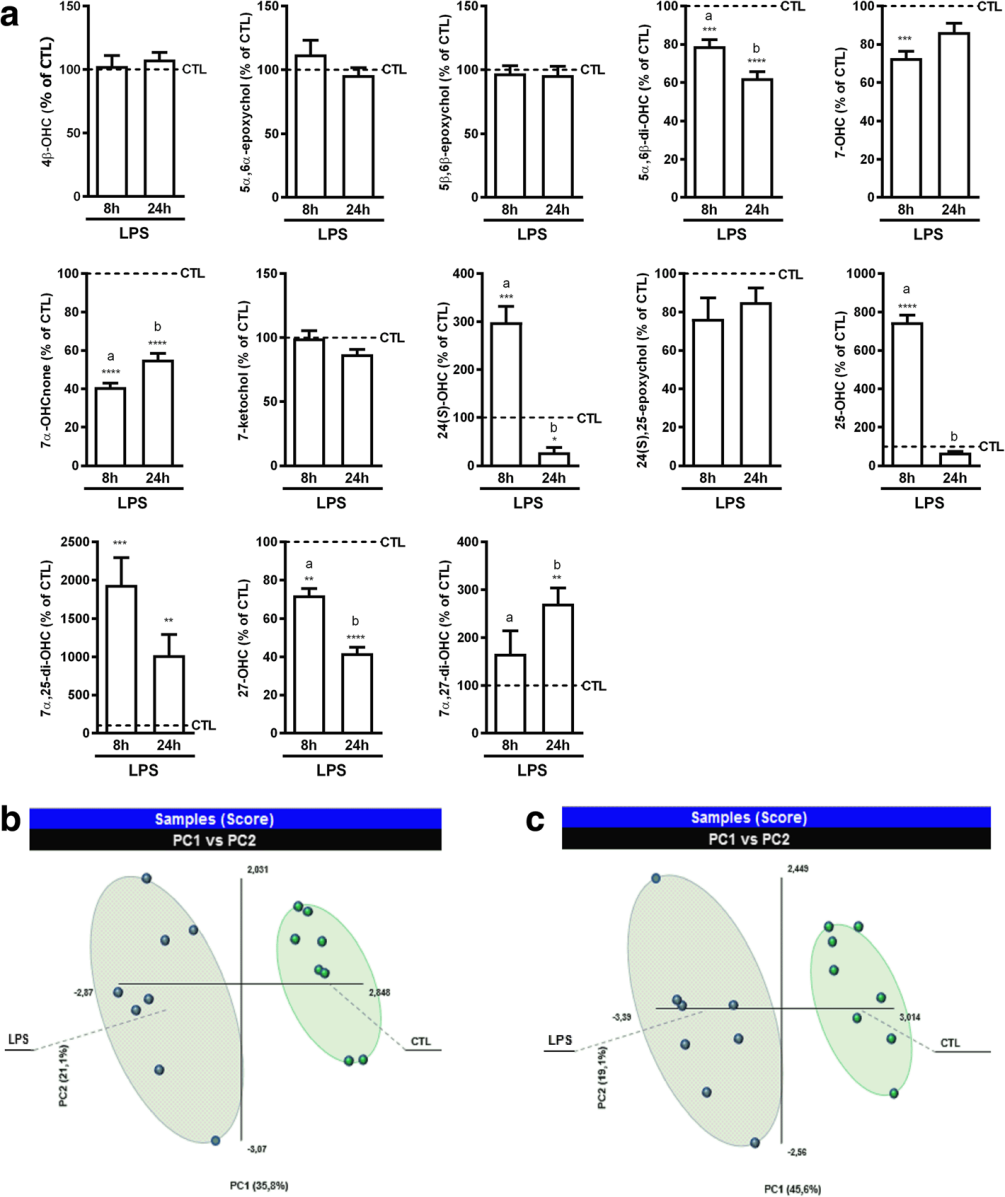
Effect of LPS activation of primary co-culture of glial cells on oxysterol levels. The cells were incubated in media with 1% FBS and containing 100 ng/mL of lipopolysaccharide (LPS) or vehicle (CTL). Oxysterols were analyzed at 8 and 24 h. **a** Oxysterol levels in LPS-activated primary co-culture of glial cells in comparison to control cells. The data are expressed as the mean ± SEM in percentage of their respective controls (CTL). ****p < 0.0001; ***p < 0.001; and **p < 0.01 for LPS-activated cells versus their respective controls. The different letters (a, b) indicate differences between the time points. **b**, **c** Multivariate analysis of the variations in oxysterol levels. The results of the partial least-squares discriminant analysis (PLS-DA) are shown as score plots for the two time points analyzed: **b** 8 and **c** 24 h. Each dot on the graph represents a dish of cells. The quality of the model was assessed by the values of *R*^2^ and *Q*^2^. These values were 0.94 and 0.80, respectively, after 8 h and 0.95 and 0.91 after 24 h

In terms of LPS effect, we observed some similarities between LPS-activated BV2 cells and this primary co-culture. For instance, 5α,6α-epoxychol and 5β,6β-epoxychol levels did not vary in either condition and 27-OHC was decreased in both (Fig. 5a). However, this was not the case for the other oxysterols as we observed no variation in 4β-OHC levels and a decrease in the levels of 5α,6β-di-OHC, 7-OHC, and 7α-OHCnone, while the levels of 25-OHC, 7α,25-di-OHC, and 7α,27-di-OHC were increased in the co-culture (Fig. 5a), thus showing opposite variations to what we observed in the BV2 cells. These variations were time-dependent and more marked at the 8-h time point, with a tendency to return to control levels at 24 h. 5α,6β-di-OHC was an exception as the decrease was more marked after 24 h of incubation. 27-OHC is another exception, as its levels were still decreased at 24 h, but this can be explained by the increased levels of its metabolite 7α,27-di-OHC (Fig. 5a). Moreover, we also quantified 24(*S*)-OHC in this co-culture and found its levels to be increased at 8 h and decreased after 24 h (Fig. 5a). Here also, when looking at the overall effect of LPS-induced activation, we found that a PLS-DA analysis could discriminate between control and LPS-treated cells based on oxysterol levels as for the BV2 cells (Fig. 5b, c).

**Fig. 6:**
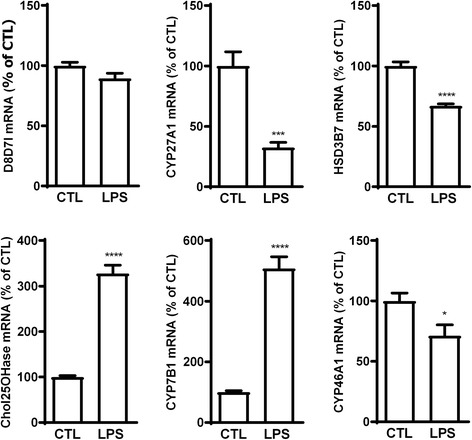
mRNA expression of oxysterol-metabolizing enzymes in LPS-activated co-culture of primary glial cells. 2.5 × 10^5^ cells were incubated with 100 ng/mL of LPS or vehicle (CTL) for 8 h. mRNA was extracted and RT-qPCR performed for CYP27A1, cholesterol 25 hydroxylase (Chol25OHase), HSD3B7, CYP7B1, and the catalytic subunit of cholesterol epoxyde hydrolase (D8D7l). The data are expressed as the mean ± SEM in percentage of their respective CTL. ****p < 0.0001 and ***p < 0.001 for LPS-activated cells versus CTL

In order to see if some of these variations in oxysterol levels could be explained by alterations in enzyme expression, we analyzed mRNA expression of the enzymes potentially involved in these alterations (Fig. 6). As most of the variations were observed at 8 h with a return to normal at 24 h, we assessed mRNA expression of the enzymes following 8 h of activation with LPS. Here also, the changes in enzyme mRNA expression support some of the variations in oxysterol levels, such as the decreased expression of CYP27A1 and HSD3B7 that support the decreased levels of 27-OHC and 7α-OHCnone, respectively (Figs. 5a and 6). Moreover, the increased expression of Chol25OHase and CYP7B1 are in line with the increased levels of 25-OHC, 7α,25-di-OHC, and 7α,27-di-OHC (Figs. 5a and 6). However, here again the expression of D8D7I does not explain the levels of 5α,6β-di-OHC.

Differences between what we observed in the BV2 cells and in the primary co-culture could be due to the limitation of using BV2 cells, but they could also reflect the interplay between microglia and other glial cells such as astrocytes. Nevertheless, these results show that LPS-induced activation leads to alterations in oxysterol levels. This further suggests that oxysterols could be important players in the pathophysiology of neuroinflammation.

### Effect of oxysterols on LPS-activated primary co-culture of microglia and astrocytes

**Fig. 7:**
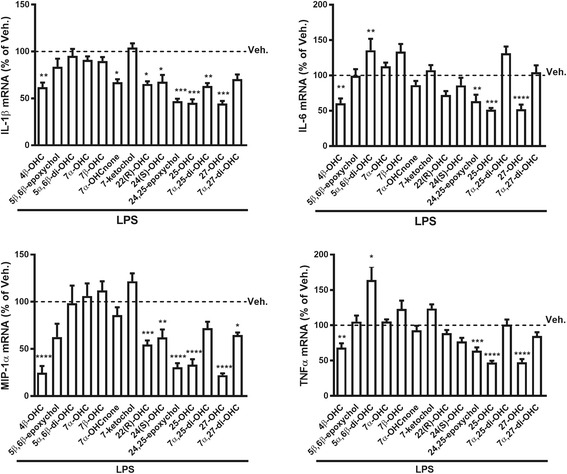
Oxysterols’ effects on mRNA expression of pro-inflammatory markers in LPS-activated co-culture of primary glial cells. 2.5 × 10^5^ cells were incubated with 10 μM of each oxysterol or vehicle (DMSO, Veh.). LPS (100 ng/mL) was added an hour later for 8 h. mRNA was extracted, and RT-qPCR performed. The data are expressed as the mean ± SEM in percentage of vehicle (Veh.) ****p < 0.0001; ***p < 0.001; **p < 0.01; and *p < 0.05 vs Veh

To see whether oxysterols could modulate glial cell activation, we assessed the effect of different oxysterols on the LPS-activated co-culture of primary glial cells. As previously reported, to evaluate the activation state of these cells, we analyzed the mRNA expression of pro-inflammatory cytokines (IL-1β, IL-6, and TNF-α) as well as the chemokine MIP-1α [27]. With the exception of 4β-OHC, the oxysterols oxidized on the sterol backbone generally had no effect on LPS-induced expression of these pro-inflammatory markers (Fig. 7). This oxysterol has been shown to activate LXRα and LXRβ in vitro [56]; however, knowledge remains scarce regarding its implication in the context of inflammation. Our data suggest a possible bioactivity of this oxysterol in activated glial cells, but further studies will be necessary to elucidate its possible implication in neuroinflammation as well as the potential pathway(s) involved. On the contrary, the oxysterols oxidized on the side chain generally decreased LPS-induced mRNA expression of IL-1β and MIP-1α, but only 24(*S*),25-epoxychol, 25-OHC, and 27-OHC decreased LPS-induced mRNA expression of IL-6 and TNF-α (Fig. 7). These side chain-oxidized oxysterols share with 22(*R*)-OHC the ability to bind and activate LXRα, LXRβ as well as RORγ [18, 57]. However, these oxysterols are also able to bind to additional receptors (e.g., 27-OHC binds to ERα [58] and GPR17 [23] while 25-OHC binds to ERα [59], Smo [60], and GPR183 [20, 61]), thus increasing the complexity of determining the actual pathway responsible for their effects. However, some of these oxysterols have not been studied in inflammatory settings before and, although preliminary in nature, these data could point towards a general regulatory role of oxysterols in neuroinflammation.

#### Variation of oxysterol levels in LPS-induced inflammation of the central nervous system

As we previously reported [31], acute systemic LPS-induced inflammation does not only affect the periphery but also leads to central nervous system (CNS) neuroinflammation. The latter is characterized by an increase of pro-inflammatory cytokines and chemokines. To assess whether oxysterol levels are also affected in such a model, we induced inflammation in C57BL/6 mice by i.p. administration of LPS (300 μg/kg) and sacrificed the mice 4 or 8 h later. To ascertain the presence of inflammation, we measured mRNA expression of IL-6 and TNF-α, both in the brain and spinal cord. We observed a strong increase in the expression of these two cytokines at 4 h, with a decrease and tendency to normalize at 8 h (Additional file 1: Figure S3). This is consistent with this model being an acute inflammation. We then measured the oxysterol levels in the brain and the spinal cord. In animals treated with the vehicle we found, consistent with the literature, that 24(*S*)-OHC was the most abundant oxysterol (Additional file 1: Figure S4A, B).

**Fig. 8:**
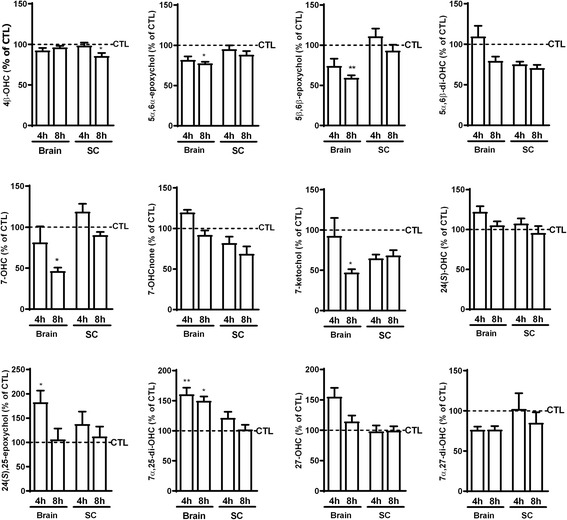
Effect of LPS-induced inflammation on oxysterol levels in the CNS compared to CTL mice. Seven mice per group were treated with LPS (300 μg/kg) or vehicle (CTL) and sacrificed after 4 or 8 h. The data are expressed as the mean ± SEM in percentage of the respective controls (CTL). **p < 0.01 and *p < 0.05 vs CTL

In the brain, we observed for the two most studied oxysterols in the context of neuropathology (i.e., 24(*S*)-OHC and 27-OHC) [62–64] only a trend to increase after 4 h (Fig. 8). However, when looking at the other oxysterols, variations were detected in inflammatory conditions. Indeed, 5α,6α-epoxychol, 5β,6β-epoxychol, 7-OHC, and 7-ketochol were decreased after 8 h of LPS administration. 7α,25-di-OHC showed an increase already after 4 h of LPS administration which was also sustained at the 8 h time point (Fig. 8). Another well-described oxysterol in the CNS, 24(*S*),25-epoxychol [65, 66] presented the same profile as 24(*S*)-OHC and 27-OHC, an increase after 4 h and normalization after 8 h (Fig. 8). In the spinal cord, the LPS-induced inflammation resulted in no major change in oxysterol levels (Fig. 8), despite the presence of a similar inflammation (Additional file 1: Figure S3B). This further supports the fact that variations in oxysterol levels are tissue-dependent even within the CNS.

In the brain, the data were in contrast to the variations observed in the BV2 cells. Indeed, many oxysterols oxidized on the sterol backbone (5α,6α-epoxychol, 5β,6β-epoxychol, 5α,6β-di-OHC, 7-OHC, and 7-ketochol) were decreased after 8 h while those oxidized on the lateral chain were rather increased. These variations are more in line with what we observed in the primary co-culture of microglia and astrocytes (Fig. 5). Besides, some oxysterols oxidized on the sterol backbone, such as 7-ketochol and 7-OHC, showed cytotoxicity when incubated with cells of the CNS [56, 67]. Therefore, the decrease of these oxysterols in the context of a systemic inflammation induced by LPS could suggest a protective mechanism to reduce their toxicity.

As oxysterols are mainly transformed into bile acids in the liver where many cytochromes P450 involved in oxysterol metabolism are expressed, we thought also to quantify oxysterols in this organ in this study. Profiles (Additional file 1: Figure S4C) were different in comparison to those observed in the brain. Nevertheless, in an interesting manner, 7α,25-di-OHC preserved the same profile as in the brain (Additional file 1: Figure S5). 25-OHC as well as 7α,27-di-OHC levels were also increased in LPS mice in comparison to control group. The epoxycholesterols (5α,6α- and 5β,6β-epoxychol) evolved in opposite manner in comparison to the brain after 8 h following LPS administration (Additional file 1: Figure S5). Moreover, 7α-OHCnone which is the first intermediate formed from oxysterols (7α-OHC) in the classical pathway of bile acid synthesis was decreased in this experiment after 4 or 8 h of LPS administration (Additional file 1: Figure S5).

#### Variation of oxysterol levels in experimental autoimmune encephalomyelitis

Several studies in multiple sclerosis (MS) patients suggest that cholesterol homeostasis is disturbed in MS with changes in oxysterol levels, mainly 24-OHC and 27-OHC, in the serum and cerebrospinal fluid (CSF) of MS patients [68–72]. However, much less is known about how other oxysterol levels are affected in MS or how MS influences oxysterol levels in the brain. The latter is of course difficult to do in human patients, and few studies have examined this aspect in animal models of MS. One study found increased circulating levels of 24-OHC and 27-OHC even before the onset of clinical signs in the EAE model, leading the authors to suggest that these two oxysterols could be used as potential biomarkers for MS [73].

**Fig. 9:**
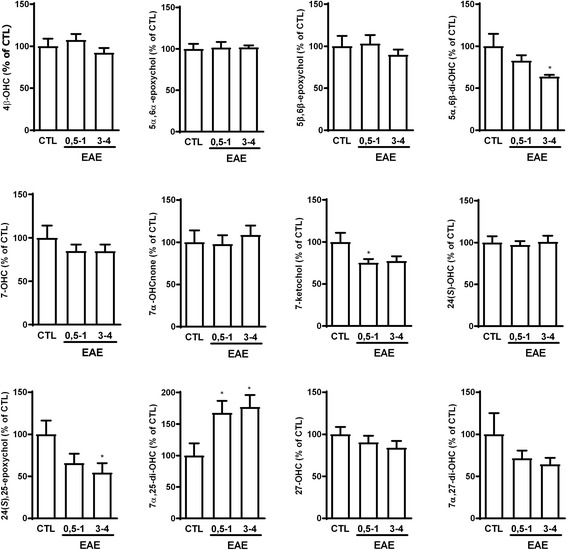
Effect of experimental autoimmune encephalomyelitis (EAE) on brainstem oxysterol levels. Mice were injected with either CFA (CTL) or MOG35-55 emulsified in CFA (EAE) followed by pertussis toxin. EAE mice were sacrificed either at a clinical score of 0.5–1 or at a clinical score of 3–4. Control mice were concomitantly sacrificed. The data are expressed as the mean ± SEM in percentage of the controls (CTL). *p < 0.05 vs CTL

Here, we measured oxysterol levels in the brainstem of mice with EAE. To have an idea if variations would be similar at different stages of the disease, we sacrificed EAE mice when the clinical score was 0.5–1 or 3–4. We found a decrease in the levels of 5α,6β-di-OHC, 7-ketochol, and 24(*S*),25-epoxychol while the levels of 7α,25-di-OHC were increased, notably in mice with a clinical score of 3 or 4 compared to control mice (Fig. 9). 7α,25-di-OHC was proposed to promote encephalitogenic CD4+ T cells migration and thus to play a pro-inflammatory role in CNS pathologies [8]. Indeed, in the same study, the authors found that deletion of Ch25h, responsible for the production of 25-OHC and its metabolite 7α,25-di-OHC, attenuates EAE disease course in mice [8]. However, this is probably not the case for all oxysterols as activation of LXRs, with 25-OHC or synthetic ligands, was shown to promote myelin gene expression and oligodendrocyte maturation in primary mixed glial cells and to promote remyelination in cerebellar organotypic cultures [74].

## Conclusions

In this study, we showed the impact of activation of BV2 cells and co-culture of primary microglia and astrocytes on oxysterol levels. Using these in vitro models, we showed that a M1-polarization drastically alters oxysterol levels and that several oxysterols were actually able to decrease LPS-induced cell activation. Our data suggest that inflammation may alter oxysterol levels beyond the usual suspects (e.g., 24(*S*)-OHC and 27-OHC) classically studied in the context of neurological diseases, thus supporting the interest of our larger screening approach of the oxysterome. Indeed, alterations in this oxysterome or in the “homeostatic” oxysterol profiles could be responsible for, or implicated in, a certain pathology, as opposed to the increased or decreased levels of a single oxysterol. Moreover, as oxysterols have several targets and may exert somewhat opposing effects in the same setting, more studies will be necessary to unravel the role of oxysterols in neuroinflammation.

Although our methodology allowed for studying the effect of cell activation/inflammation on numerous oxysterol levels and conversely the effect of oxysterols on cell activation, clearly, the mechanisms supporting these effects still remain to be clarified. Further studies will be necessary to determine the mechanisms by which active oxysterols control the expression of the pro-inflammatory markers. This is undeniably an active field of research with an increasing number of molecular targets identified for oxysterols. Overall, our study should contribute to a better understanding of the interplay between oxysterols and neuroinflammation.

## Additional file

Additional file 1: **Figure S1.** Activation of BV2 cells and oxysterol levels in control BV2 cells. (A) 2.5 × 10^5^ cells were incubated with vehicle (CTL) or LPS (100 ng/mL) for the indicated time points. mRNA was extracted and RT-qPCR performed for IL-1β, IL-6, and TNF-α. The data are expressed as the mean ± S.E.M in percentage of the respective CTL. ***p < 0.001, **p < 0.01, and *p < 0.05 vs CTL. (B) Oxysterols were quantified in BV2 cells incubated without LPS. The data are expressed as the mean ± S.E.M in pmol/10*10^6^ cells. (C) 2.5 × 10^5^ cells were incubated with vehicle (CTL) or 10 U/mL of IL-4 for the indicated time points. mRNA was extracted and RT-qPCR performed for Arg1 and CD206. The data are expressed as the mean ± S.E.M in percentage of the respective CTL. **Figure S2.** LPS-induced activation of primary co-culture of astrocytes and microglia and oxysterol levels in control cells. (A) 2.5 × 10^5^ cells were incubated with LPS (100 ng/mL) or vehicle (CTL) for 8 h. mRNA was extracted and RT-qPCR performed for IL-1β, IL-6, and TNF-⍺. The data are expressed as mean ± S.E.M in percentage of CTL set at 100. ****p < 0.0001 and ***p < 0.001 vs CTL. (B) Oxysterols were quantified in co-culture of primary microglia and astrocytes incubated without LPS. The data are expressed as the mean ± S.E.M in pmol/10 × 10^6^ cells. **Figure S3.** mRNA expression of pro-inflammatory markers in (A) the brain and (B) spinal cord of mice with LPS-induced inflammation in comparison to control mice. Mice (seven per group) were treated with LPS (300 μg/kg) or vehicle (CTL) and sacrificed after 4 or 8 h. mRNA was extracted and RT-qPCR was performed for IL-6 and TNF-α. The data are expressed as the mean ± S.E.M in percentage of CTL set at 100. ***p < 0.001 vs CTL. **Figure S4.** Oxysterol levels in CTL mice. Oxysterols were analyzed in seven control mice, in the brain (A), the spinal cord (B), and the liver (C). The data are expressed as the mean ± S.E.M in pmol/g of tissue. **Figure S5.** Effect of LPS-induced inflammation on oxysterol levels in the liver in comparison to CTL mice. Seven mice per group were treated with LPS (300 μg/kg) or vehicle (CTL) and sacrificed after 4 or 8 h. The data are expressed as mean ± S.E.M in percentage of CTL. **p < 0.01 and *p < 0.05 vs CTL. (PDF 103 kb)

## Abbreviations

BHT: Butylated hydroxytoluene
CD: Cluster of differentiation
Chol25OHase: Cholesterol 25 hydroxylase
CSF: Cerebrospinal fluid
CXCR2: C-X-C chemokine receptor type 2
CYP: Cytochrome P450
D8D7I: 3beta-hydroxysterol-Delta8-Delta7-isomerase
EAE: Experimental autoimmune encephalomyelitis
Epoxychol: Epoxycholesterol
ERα: Estrogen receptor α
GPR: G protein-coupled receptor
HSD3B7: Hydroxy-delta-5-steroid dehydrogenase, 3 beta- and steroid delta-isomerase 7
IL: Interleukin
Ketochol: Ketocholesterol
LPS: Lipopolysaccharides
LXR: Liver X receptor
MOG: Myelin oligodendrocyte glycoprotein
OHC: Hydroxycholesterol
OHCnone: Hydroxycholestenone
PTX: Pertussis toxin
ROR: Retinoic acid-related orphan receptor
ROS: Reactive oxygen species
Smo: Smoothened
TNF-α: Tumor necrosis factor α
