# Supplementary material for: Oxysterol levels and metabolism in the course of neuroinflammation: insights from in vitro and in vivo models
Source: J Neuroinflammation. 2018 Mar 9;15:74. doi: 10.1186/s12974-018-1114-8 (PMC5845224; doi:10.1186/s12974-018-1114-8)
Supplement: Supplementary file 1 — Figure S1. Activation of BV2 cells and oxysterol levels in control BV2 cells. (A) 2.5 × 10^5^ cells were incubated with vehicle (CTL) or LPS (100 ng/mL) for the indicated time points. mRNA was extracted and RT-qPCR performed for IL-1β, IL-6, and TNF-α. The data are expressed as the mean ± S.E.M in percentage of the respective CTL. ***p < 0.001, **p < 0.01, and *p < 0.05 vs CTL. (B) Oxysterols were quantified in BV2 cells incubated without LPS. The data are expressed as the mean ± S.E.M in pmol/10*106 cells. (C) 2.5 × 10^5^ cells were incubated with vehicle (CTL) or 10 U/mL of IL-4 for the indicated time points. mRNA was extracted and RT-qPCR performed for Arg1 and CD206. The data are expressed as the mean ± S.E.M in percentage of the respective CTL. Figure S2. LPS-induced activation of primary co-culture of astrocytes and microglia and oxysterol levels in control cells. (A) 2.5 × 10^5^ cells were incubated with LPS (100 ng/mL) or vehicle (CTL) for 8 h. mRNA was extracted and RT-qPCR performed for IL-1β, IL-6, and TNF-⍺. The data are expressed as mean ± S.E.M in percentage of CTL set at 100. ****p < 0.0001 and ***p < 0.001 vs CTL. (B) Oxysterols were quantified in co-culture of primary microglia and astrocytes incubated without LPS. The data are expressed as the mean ± S.E.M in pmol/10 × 106 cells. Figure S3. mRNA expression of pro-inflammatory markers in (A) the brain and (B) spinal cord of mice with LPS-induced inflammation in comparison to control mice. Mice (seven per group) were treated with LPS (300 μg/kg) or vehicle (CTL) and sacrificed after 4 or 8 h. mRNA was extracted and RT-qPCR was performed for IL-6 and TNF-α. The data are expressed as the mean ± S.E.M in percentage of CTL set at 100. ***p < 0.001 vs CTL. Figure S4. Oxysterol levels in CTL mice. Oxysterols were analyzed in seven control mice, in the brain (A), the spinal cord (B), and the liver (C). The data are expressed as the mean ± S.E.M in pmol/g of tissue. Figure S5. Effect of LPS-induced inflammation on [file 12974_2018_1114_MOESM1_ESM.pdf]

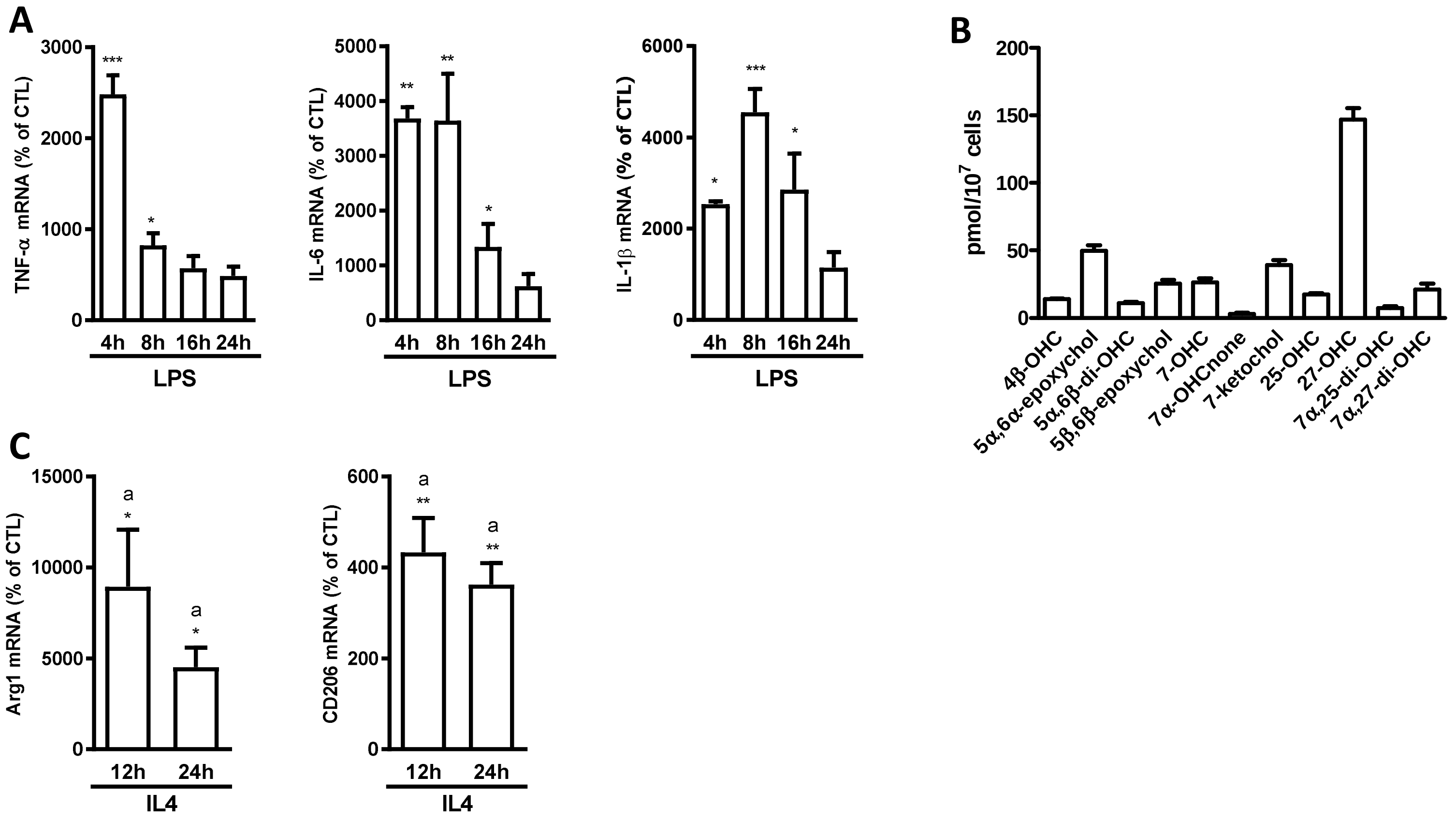

Supplementary figure 1: **Activation of BV2 cells and oxysterol levels in control BV2 cells**
(A) 2,5 * 10^5 cells were incubated with vehicle (CTL) or LPS (100 ng/mL) for the indicated time points. mRNA was extracted and RT-qPCR performed for IL-1β, IL-6 and TNF-1α. Data are expressed as the mean ± S.E.M in % of the respective CTL. *** p < 0,001; ** p < 0,01; * p < 0,05 vs CTL. **(B)** Oxysterols were quantified in BV2 cells incubated without LPS. Data are expressed as the mean ± S.E.M in pmol/10*10^6 cells. **(C)** 2,5 * 10^5 cells were incubated with vehicle (CTL) or 10 U/mL of IL-4 for the indicated time points. mRNA was extracted and RT-qPCR performed for Arg1 and CD206. Data are expressed as the mean ± S.E.M in % of the respective CTL.

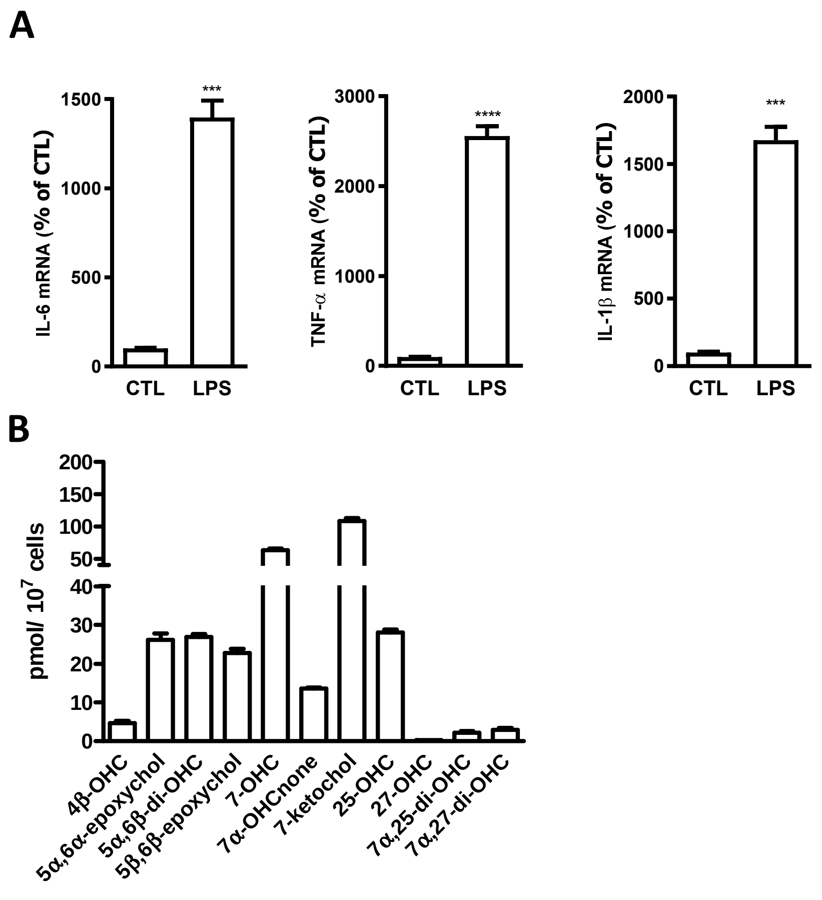

Supplementary Figure 2: **LPS-induced activation of primary co-culture of astrocytes and microglia and oxysterol levels in control cells** **(A)** 2,5* 10^5 cells were incubated with LPS (100 ng/mL) or vehicle (CTL) for 8 hours. mRNA was extracted and RT-qPCR performed for IL-1β, IL-6 and TNF-α. Data are expressed as mean ± S.E.M in % of CTL. **** p < 0,0001; *** p < 0,001 vs CTL. **(B)** Oxysterols were quantified in co-culture of primary microglia and astrocytes incubated without LPS. Data are expressed as the mean ± S.E.M in pmol/10*10^6 cells.

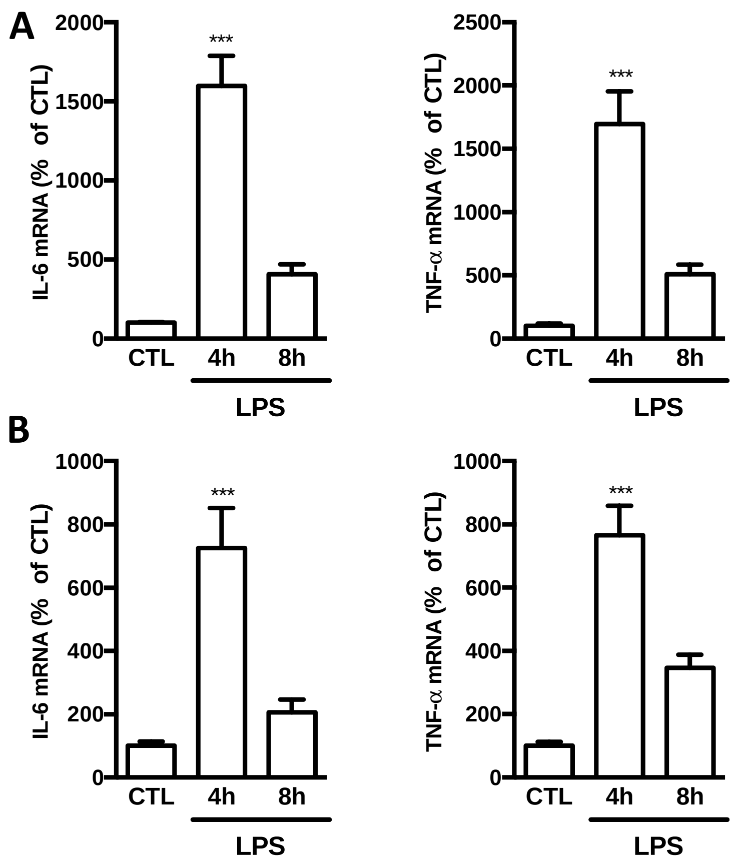

Supplementary Figure 3: **mRNA expression of pro-inflammatory markers in (A) the brain and (B) spinal cord of mice with LPS-induced inflammation in comparison to control mice**. Mice (7 per group) were treated with LPS (300 µg/kg) or vehicle (CTL) and sacrificed after 4 or 8 hours. mRNA was extracted and RT-qPCR was performed for IL-6 and TNF-α. Data are expressed as the mean ± S.E.M in % of CTL set at 100. *** $p < 0,001$ vs CTL.

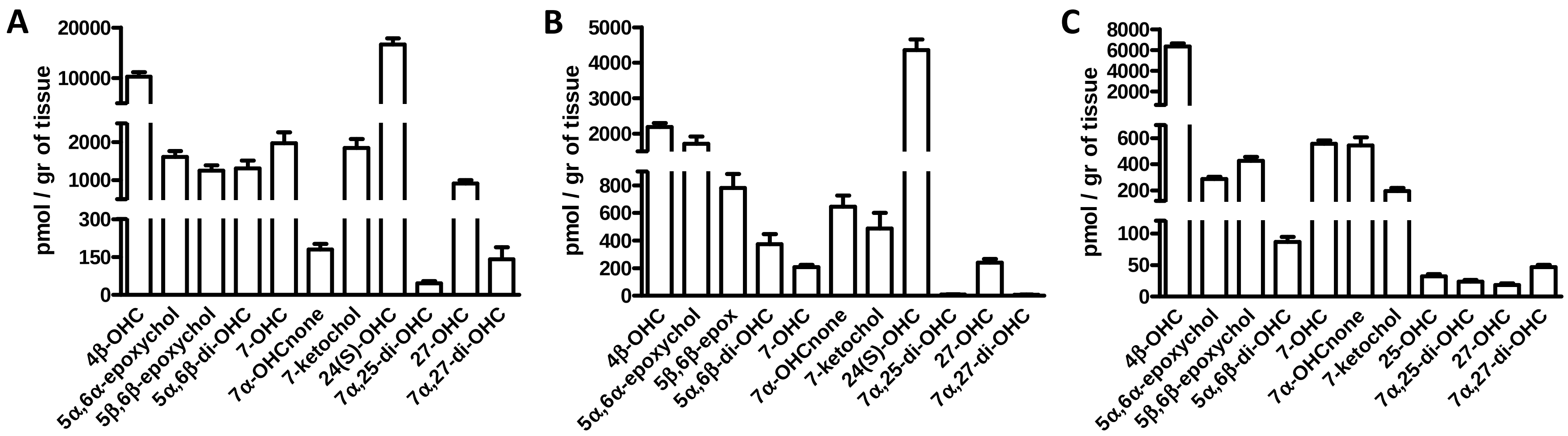

Supplementary Figure 4: **Oxysterol levels in CTL mice**. Oxysterols were analysed in 7 control mice, in the brain (**A**), the spinal cord (**B**) and the liver (**C**). Data are expressed as the mean ± S.E.M in pmol / gram of tissue.

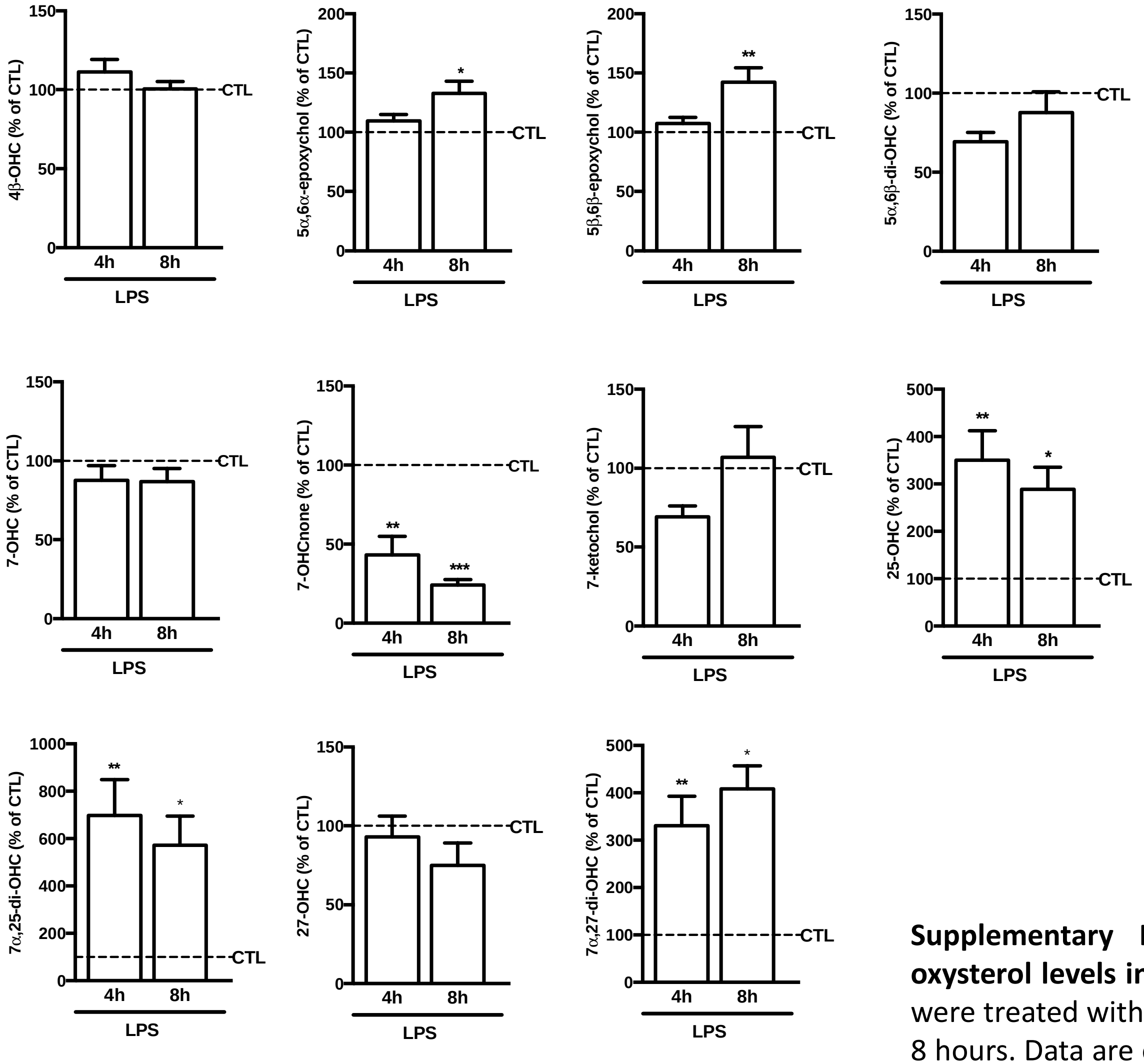

Supplementary Figure 5: **Effect of LPS-induced inflammation on oxysterol levels in the liver in comparison to CTL mice**. 7 mice per group were treated with LPS (300 µg/kg) or vehicle (CTL) and sacrificed after 4 or 8 hours. Data are expressed as mean ± S.E.M in % of CTL. ** p < 0,01 * p < 0,05 vs CTL.
